# Complete Genome Sequence of *Streptococcus pyogenes* Strain JMUB1235 Isolated from an Acute Phlegmonous Gastritis Patient

**DOI:** 10.1128/genomeA.01133-16

**Published:** 2016-10-20

**Authors:** Shinya Watanabe, Teppei Sasahara, Naoshi Arai, Kazumasa Sasaki, Yoshifumi Aiba, Yusuke Sato’o, Longzhu Cui

**Affiliations:** a Division of Bacteriology, Department of Infection and Immunity, Jichi Medical University Hospital, Yakushiji, Shimotsuke-shi, Tochigi Prefecture, Japan; b Division of Gastroenterology, School of Medicine, Jichi Medical University, Yakushiji, Shimotsuke-shi, Tochigi Prefecture, Japan; c Division of Microbiology Laboratory, Jichi Medical University Hospital, Yakushiji, Shimotsuke-shi, Tochigi Prefecture, Japan

## Abstract

Acute phlegmonous gastritis is an uncommon endogenous bacterial gastritis presenting with a high mortality rate. Here, we report the complete genome sequence of an *emm*89 *Streptococcus pyogenes* strain, JMUB1235, which is the causative agent of acute phlegmonous gastritis.

## GENOME ANNOUNCEMENT

Acute phlegmonous gastritis (APG) is a rare and rapidly progressive endogenous bacterial infection with high mortality (1). The most frequent causative bacteria of APG are hemolytic streptococci, particularly group A streptococci (GAS) (2, 3). GAS is a group of important human pathogens that causes a wide range of infections from local skin infections to life-threatening severe systemic diseases, including streptococcal toxic shock syndrome and necrotizing fasciitis. Accordingly, there have been many studies of GAS genomes relevant to the ordinary infections but none on whole-genome sequencing of the GAS strain that causes APG. Here, we report the whole-genome sequence of *Streptococcus pyogenes* strain JMUB1235 isolated from an APG patient in Jichi Medical University Hospital, Japan, in 2016.

Bacterial culture and DNA extraction were performed as previously described (4, 5). A mate-pair sequencing library from whole-genome DNA was prepared using the Nextera mate-pair sample preparation kit (Illumina, Inc., San Diego, CA, USA) without size selection. Sequencing was performed using the Illumina MiSeq platform (2 × 301 bp) with the MiSeq reagent kit version 3 (Illumina, Inc.), which generated 1,155,944 paired-end reads. After quality trimming using the FASTQ toolkit version 2.0.0 with a quality level of 30, a total of 1,055,944 high-quality reads were assembled with the Velvet *de novo* assembly version 1.2.10 algorithm into contigs and a scaffold. The resulting assembly comprised 21 contigs, 10 of which were linked to a 1,733,042-bp scaffold close to the expected size of the *S. pyogenes* genome. Ten persisting gaps were filled by gap-spanning PCR, followed by Sanger sequencing using an ABI3130xl genetic analyzer (Applied Biosystems, Carlsbad, CA, USA) to generate a single circular genome. Gene extraction and annotation were performed using the Microbial Genome Annotation Pipeline (http://www.migap.org).

*S. pyogenes* strain JMUB1235 harbors a single circular genome of 1,741,982 bp (G+C content, 39.2%) and no plasmid. A total of 1,717 coding sequences, 57 tRNA genes, and 15 rRNA genes were identified. JMUB1235 is an *emm*89 strain; it is increasingly recognized as a leading cause of the disease worldwide and is reportedly the dominant strain in the United Kingdom (6). The JMUB1235 lost hyarulonic acid capsular synthase gene (*hasABC*) was similar to the most closely related genome of the M89 epidemic strain MGAS27061, showing an acapsular phenotype (7). On the other hand, compared with MGAS27061, JMUB1235 had polymorphisms in two membrane proteins (SclA and M protein), with amino acid identities of 78% and 87%, respectively, and carried an intact negative gene regulator, *csrSR* (synonym*, covSR*). In addition, two clustered regularly interspaced short palindromic repeat (CRISPR) candidates were identified in JMUB1235 using the CRISPR Finder tool (http://crispr.i2bc.paris-saclay.fr/). CRISPR-1 was identical in two strains, while the number of spacers in CRISPR-2 differed, occurring five and eight times in the genomes of JMUB1235 and MGAS27061, respectively. Overall, the genome analysis of strain JMUB1235 can provide insight into understanding the pathological process of APG caused by GAS.

### Accession number(s).

The complete genome sequence of strain JMUB1235 has been deposited in the DDBJ GenBank under the accession number AP017629.
